# Nursing Conditions in Pay Hospitals

**Published:** 1920-04-03

**Authors:** 


### THE MATRONS' AND SISTERS' DEPARTMENT.

### NURSING CONDITIONS IN PAY HOSPITALS.

The new movement in favour of pay-wards and pay-hospitals requires very careful watching in the interests of nurses, no less than of the patients. It is avowedly a campaign on behalf of that section of the public which lies between the hospital and the nursing-home clientèle, and will impinge on both. That is to say that many persons reluctantly compelled in the past to accept free treatment will prefer to pay some part, at any rate, of the expense when opportunity offers. And many who have hitherto with considerable difficulty found money to pay private nurses or private nursing-home fees, will gravitate towards institutions which offer the same advantages at a lower cost.

What nurses want to know is the extent to which this new system is likely to affect their earning powers and prospects. Truth to tell, we are not satisfied that they have in the past fared particularly well under the pay-ward system, and so far as nursing goes, the patients have often fared very hardly indeed. Pay-wards exist in many special and in a few general hospitals. Under the ordinary nursing administration these wards are largely served by probationers, but inasmuch as the untrained women work apart in single rooms or small wards, they often receive far less supervision than they would otherwise do. There ought, of course, to be a sister whose services are reserved exclusively for the pay-wards, but this is by no means always the case.

The use of probationers in their first year among paying patients for the performance of any nursing functions appears to us entirely indefensible. This abuse is one of the standing complaints against ill-managed nursing-homes, and in any measure designed for the regulation of these establishments the most important proviso should surely be one for the exclusion of any but fully-trained nurses. If municipal institutions for paying patients introduce the unskilled hand under any pretext whatever, injury will be done to three separate individuals. The so-called probationer will be wasting time which she ought to be spending in an institution capable of affording her a recognised certificate. The patient who relies upon being tended by a trained nurse will be deceived and exposed to possible danger. And the trained private nurse who would have attended the case had the patient remained at home will have lost her fee.

The private nurse possibly stands, in any case, to find her occupation curtailed by the extension of the pay-ward system. It is among patients who can barely afford to pay nursing fees that the worst abuses of private nursing occur, over-long hours, reduced fees, insanitary conditions of living. But be that as it may, the fact remains that there is a large body of women who depend for their daily bread on these private patients. If the pay-ward system tends to deprive them of occupation, they should at least be offered priority of employment under that system.

In planning pay-hospitals or converting old workhouses and infirmaries to this use, the position of the nurses ought to be safeguarded. A resident superintendent of nurses of high standing, adequately paid, is, of course, the first requirement, and the new system should provide good posts for some who now tread to their destruction along the path of private-venture nursing-homes. The nursing staff should consist of fully-trained women, engaged on the basis of a forty-eight hour week, and their salary should approximate to what they might reasonably expect to earn as private nurses, due regard being had to the greater permanence and security of the engagement. The greater leisure which the reduced working hours afford makes it desirable that the staff, who would be in a widely different position to that of nurses in training, should enjoy complete liberty of action when not on duty. It would materially hamper the construction of municipal pay-hospitals were it necessary to construct a £50,000 Nurses' Home for each. Private enterprise might be encouraged to run hostels on a smaller scale, for the accommodation of the nurses, and such as preferred it might make their home near at hand with relatives.

Under such conditions the interests of all concerned would be safeguarded. The patients would secure the benefit of skilled nursing. No probationers would be lured to sacrifice a year or two out of their best pupil period, while the nursing sisters employed would enjoy highly satisfactory conditions and gain experience of a very special character. They would then find no reason to regret the decline of private nursing.

